# Using Social Media to Preserve Consumers’ Awareness on Food Identity in Times of Crisis: The Case of Bakeries

**DOI:** 10.3390/ijerph18126251

**Published:** 2021-06-09

**Authors:** Francesc Fusté-Forné, Nela Filimon

**Affiliations:** 1 Department of Business, University of Girona, 17071 Girona, Spain; 2 Serra Húnter Fellow, Department of Business, University of Girona, 17071 Girona, Spain; nela.filimon@udg.edu

**Keywords:** branding strategies, crisis management, food experience, food marketing, food tourism, health and food, Spain

## Abstract

Departing from the understanding of food tourism in urban environments, this research analyses the brand engagement of bakeries during the COVID-19 lockdown period, and the first stages of the de-escalation process. A mixed-methods study is designed to analyze the case of six selected bakeries in Catalonia (Spain). Drawing on data obtained from semi-structured interviews (N = 6) and a visual content analysis of the businesses’ social media promotion in Instagram (N = 638), results show the performance of bakeries during pandemic times, where a change in production and consumption behaviors is observed and takeaway and delivery helped them to survive. In particular, their social media promotion in Instagram also revealed how bakeries have managed this difficult situation and kept a close relationship with customers, standing up as a symbol of resilience against the odds and contributing to preserve customers’ awareness on food and health, and the city’s identity, through digital branding strategies that communicate messages around bread and pastry foods (the product), the shop and the workshop (the place), and both the employees and the customers (the people).

## 1. Introduction

Food consumption is a critical element of tourism industry [1] and food is a motivation factor for traveling [2]. According to previous reports, eight out of ten visitors are influenced by food attractions when they select a destination [3]. Furthermore, food and gastronomy account for 40% of the world’s tourism expenditure [3], where data from countries like Spain reveal that budget of food tourists is 20% higher than of other tourists [4]. Food tourism is a very important segment of the industry, and it is described as “the pursuit and enjoyment of unique and memorable food and drink experiences, both near and far” [5] (p. 12). Among the variety of activities and practices classified under the understanding of food tourism, bread plays a significant role, the proof of this being the so-called Spanish Route of Good Bread, a national-level competition organized every year with the purpose of selecting the top eighty Spanish bakeries, among the bakeries that participate in the regional routes of good bread competitions. In this setting, top bakeries are selected based on the quality of the (traditional) bread elaborated, the presentation of the product in the store and the customer service [6]. Bread routes have inspired many local and regional tourism initiatives which use bread to show visitors the route followed by the wheat from the fields to the table, promoting at the same time territories with a strong local identity, rich gastronomic heritages and attractive landscapes. In this fashion, this paper analyses bakeries as an example of food leisure and tourism attraction, which are a significant element of city food landscapes [7] from a local perspective. In a context where only limited domestic travel was occurring because of the pandemic situation, bakeries are understood as a crucial ingredient of food tourism systems [8], in this case applied to urban environments.

Earlier in 2020 the rapid spread of the COVID-19 led to a quick closure of tourism worldwide (see, for example, [9,10,11,12]). In particular, in Spain, where the tourism sector plays a strategic role, representing 12.3% of the GDP and 12.7% of the employment [13], the impact of the COVID-19 pandemic translated into a 77.30% drop in the international tourists’ arrivals (or more than 64 million tourists), in 2020 compared to 2019 [14]. Similarly, according to the Spanish National Institute of Statistics (INE), hotel accommodation has decreased by 80.4% and air transport by 80.1% [14]. Concerning the economic impact of the pandemic, total expenditure by international tourists who visited Spain in 2020 reached 19,739 million euros, that is, 78.52% less than in 2019 ([15], see also [16]). Although the start of the summer brought a relaxation of lockdown measures, ease of travel restrictions and reopening of hospitality services, in 2020 the pandemic largely affected not only tourism, but also other sectors such as food and beverages industries, where the impact was nevertheless lower [17]. A good example of this trend is the agricultural and fishing sector, whose overall contribution to GDP in 2020 has registered a rise of 5.3%, compared to 2019, when it fell by 2.3% compared to 2018 [18].

While the overall impact of pandemics in gastronomic sector was large, it is important to highlight the significant change observed in the Spaniards’ food consumption patterns (and also most of the European and world citizens), who switched from consuming food at restaurants and other food service establishments to food consumption at home [19], and the subsequent increase of the e-commerce channel, especially during the periods of lockdown. Thus, data collected during pandemic (see [19]) showed that food expenditure in large supermarkets and food storages increased by 50% in 2020, compared to 2019, and e-commerce reached a 2.4% market share in the period March–June 2020, compared to 1.6% in 2019. The same study observes a higher dependence of the food service sector on domestic tourism, with data for 2019 unfolding as follows: Expenditure made with international cards (21%); expenditure made with Spanish cards belonging to a location different than the one of the food establishment (15%); and the remaining 64%, corresponding to cards from the same location (see [19]). This illustrates a shift towards local consumption as a symbol of sustainable consumption. According to the May Barometer of the Centre for Sociological Research [20], the online purchases of food and food products during the lockdown reached 20%, and 23.3% of the respondents indicated an increase of their usual online purchases (see also [21]).

This research understands food (i.e., bread) as a manifestation of a local culture and the local lifestyles (see, for example, [22]) and as a symbol of place [23]. Food is a key element within the construction of food image [24], which communicates a unique identity (see also [25]). Therefore food is also a critical attribute in destination marketing [26,27]. In this sense, the objective of this paper is to analyze the marketing strategy of small businesses, such as bakeries, in pandemic times. Both local stakeholders’ narratives (see [28]) and online induced images (see [29]) are used to delineate the processes of brand engagement of bakeries in Catalonia, north-eastern Spain. Previous data show that, in times of pandemic, social media platforms, such as Instagram, can be used as effective communication tools between the bakeries and their customers not only to reach new publics and build brand loyalty but also to communicate sustainable marketing practices and consumers’ responsible behaviors (see also [30]). Moreover, food “increases hedonistic experiences via digital transformations” [31] (p. 2), and the study of the role of food in marketing as part of crisis management is crucial to discuss how the promotion of food is shaped nowadays and how local food businesses are gatekeepers of a sustainable food consumption. This paper adds texture to this conversation and contributes to the understanding of the branding identity of bakeries as an urban leisure and tourism attraction.

## 2. Literature Review

This section is divided into four sub-sections. First, it reviews the concept of food tourism and defines the role of bakeries in destination management and marketing. This is expanded to understand their significance as a food experience in destination and place branding. Later, the relationships between food consumption and digital marketing are discussed to finally delineate the importance of Instagram as an example of social media that contributes to the development of online food branding.

### 2.1. Bakeries as a Manifestation of a Food Tourism Attraction

Food is a powerful tourist attraction [23]. Moreover, according to the World Food Travel Association (WFTA), basically all travelers (93%) are food travelers [32]. Food tourism refers to the exploration of a culture through food [33]. This includes many examples of practices as previous definitions of the concept have confirmed. For example, Hall and Sharples [34] described food tourism as a journey to visit food producers or markets, attend festivals and events, and eat at restaurants and any other food activity and experience. In the last decade, a lot of research has analyzed and expanded the role of food tourism as a specific tourism system [31,35] that contributes to the process of awarding tourism value to food [36]. This provides the relationship between local food and tourism with an added value where positive food experiences lead to satisfaction and word-of-mouth [37]. These authors state that “food activities may impact satisfaction with a trip and influence a traveler’s intention to return, along with recommendations and food purchase behavior at home” [37] (p. 151). As a consequence, food must be placed at the center of destination marketing, where both everyday food consumption and memorable food experiences are included.

Du Rand, Heath and Alberts [38] highlight, for example, that “the traditional way that food experiences are offered in a destination is reflected by the promotion of restaurants. This form of food experience occurs more easily as food has to be provided to tourists, it does not involve additional effort, organization and promotional activities as for example a food festival would require” (p. 106). This is similarly applied to the case of bakeries. This present study contributes to expand the literature of marketing food tourism through the case of a specific type of food establishment.

Businesses which offer food and beverage experiences do not only deliver food and dishes, but they also promote the processes attached both to production and consumption of food (see, for example, [39]). This generates a diverse landscape of options to design food-based practices and develop strategies to attract locals and visitors who seek the discovery of a place through its food. Farmers’ markets, chef’s talks, culinary tours, all are examples of the incorporation of food into tourism, which is a prerequisite towards the development of food tourism systems. Kollenberg et al. [39] affirm that this happens in a very competitive environment, where many regions rely on food as a source of competitive advantage, which thus urges businesses and destinations to differentiate their services. This paper aims to provide additional evidence to the existing debate focused on the case of bakeries.

Food and gastronomy are also key identity markers [24,25,40]. In this sense, “food is very important to city marketing, and vise-versa. Food is part of the culture of a city or a nation, it’s part of its symbolic capital. Even so if the city doesn’t have a strong gastronomic identity. It is important because food is not only about stomachs, but also about quality of life, about meeting people, sharing experiences, sharing a taste” (Maynadier, 2014, cited in [7], p. 108). According to Kowalczyk [7], the culinary identity of a city includes different outstanding elements like bakeries.

### 2.2. Bake Your Food, Promote Your Place

Food consumption is a vital component of leisure and tourist experiences [41,42]. Food is critical in destination branding and marketing [1,23] and is useful to build a differentiation strategy (see, for example, [43,44]), where a particular food aspect is highlighted. A brand experience includes the customers’ perceptions gathered within their interactions with a firm [45]. Marketing strategies are required to focus on co-production and co-creation of experiences [46].

In 2006, Du Rand and Heath [26] revealed that, while research about the contribution of food to destination marketing was in its infancy, food tourism was already a critical element of destination marketing. In this sense, the definition of food tourism provided by Hall and Sharples [34] reveals that food tourism is put in practice drawing on a diverse range of products and services. This is a prerequisite towards the development of marketing strategies around products, events or venues. Food tourism emerges as a key element in tourism promotion [26]. Given the growing significance of food for tourists, and the rise of food tourism as a segment of cultural tourism [47] both companies and destinations need to plan effective food tourism marketing strategies [48].

Destination branding is of crucial importance to stakeholders [49], which contributes towards the promotion of a place [50]. As part of their branding strategy, a place aims to enhance their identities and uniqueness [51,52]. In this sense, destination branding is complex [53] since it depends on a wide range of factors. Food emerges as one of them [54,55]. Previous research acknowledged that a destination branding strategy should primarily rely on what locals understand as determinants of sense of place as a source of effective destination marketing [56]. Attributes of local food are useful to communicate local knowledge [57]. In this context, food is a strong manifestation of destination identity and thus represents a powerful tool towards the construction of a destination brand [58].

Previous research has demonstrated the effectiveness of food festivals as identity elements that play a prevalent role for destination branding [59,60]. As city branding also reflects the identity of a place [61], in this case an urban environment, this paper assumes that the way bakeries market themselves convey how they are as part of city food landscapes. The formation of a destination brand is an instrument to gather competitive advantage [62] and this research explores how bakeries as local stakeholders create a unique experience drawing on their branding strategy and contribute to destination branding. Previous studies acknowledged that the creation of a unique brand image also inform memorable experiences and generate positive word-of-mouth (see, for example, [41,63]), as mentioned above.

In the framework of destination branding (see, for example, [64]), recent research also highlights the role of food tourism in place branding. In this sense, the value of food experiences influences tourists’ perceptions of food tourism [65]. Value creation is critical in tourism experiences [66] where experiential value is in turn an antecedent of branding in food tourism [67]. This is especially relevant in brand experiences, where customer-firm interactions generate behavioral responses [68]. As previously acknowledged, food tourism offers a wide range of opportunities to discover a place through food consumption. In this sense, food is a crucial element of sense of place [69] and thus food tourism represents an avenue to authentic local-based sustainable experiences [23].

### 2.3. Food Consumption and Digital Marketing

The use of local food is critical to define a brand image [41,70]. According to Qu, Kim and Im [71], the process of destination branding refers to the generation of a unique image, which is also a source of differentiation, and a motivation factor for visitors [67]. This can be applied to businesses (say bakeries), where a series of factors such as service quality contribute to improve their brand image [72,73]. In a current context dominated by the rapid transmission of COVID-19, health is also an important aspect for customers in regards to their food experience and the gathering of consumption value [74]. This is pivotal in food consumption, and even more relevant nowadays. Culinary-based experiences must focus on protecting customers from health risks and secure hygiene and safety [27]. This will influence tourists’ attitude towards local food and, in turn, impact on the creation of food image. However, this approach is not explored in this paper which is focused on analyzing the bakeries’ branding during pandemic times, from a supply perspective.

At the same time, rapid evolution of social media and information and communication technologies, such as mobile technologies, have changed the traditional relationships between businesses and customers [75]. They have revolutionized branding and marketing strategies [76]. In the same line, Buhalis and Sinarta [77], affirm that “brands take advantage of technology, social media and constant connectivity to foster organic consumer engagement and interactions towards co-creating personalized customer service. Realtime service offers dynamic engagement with connected consumers. Brands in tourism and hospitality use technology to dynamically enhance consumer experience through cocreation” (p. 1).

The use of online marketing is a common avenue to reach customers. Social media is a growing platform for tourists’ decision-making [78,79]. Digital content marketing aims to increase interactions between customers and the brand, and add value to the service experience and promote long-term relationships [80,81]. According to Jefferson and Tanton [82], digital content marketing goes beyond sales to focus on communication in order to build strong emotional connections [83]. Building on Westbrook’s [84] concept of word-of-mouth transmission, here it is important to acknowledge the electronic word-of-mouth (e-WOM), which is described as “all informal communications directed at consumers through Internet-based technology related to the usage or characteristics of particular goods or services, or their sellers” ([84], cited in [85], p. 459). Businesses influence visitors on social media as an effective strategy to communicate brand identity [80].

Furthermore, social media boosts a direct interaction between brands and customers, which results in a growing trend for co-creating “value and experiences in real time” [77] (p. 565). These authors acknowledge the notion of real-time marketing “as marketing that provides personalized, individualized and contextualized products and services, based on real-time dynamic engagement with customers and co-creation of experiences, to optimize value for all stakeholders involved. Real-time marketing is propelling “nowness” or present moment service and experience” (p. 565). In this sense, “delivering the right content to the right consumer at the right time, especially on unplanned circumstances, requires a lot more than merely, observing, listening, designing, testing and creating expected service. It is caused by consumers’ unique and dynamic personas, that are all influenced by their present context and location” (p. 566). As Buhalis and Sinarta [77] (p. 575) continue, ‘the service of now’ “emerges through the ability of engaging with consumers dynamically at the present time both physically and digitally”. In a current crisis environment, which is rapidly changing and uncertain, real-time communication is essential to food-based experiences. Recent research highlighted the positive effects of adopting social media by small and medium-sized enterprises (SMEs) in a crisis situation [86]. Among the competitive advantages derived from the use of social media in crisis management (see [87]), communication in social media platforms illustrate branding responses to the COVID-19 crisis [88] and provide evidence of the place brand personality [89].

### 2.4. Social Media and Online Food Branding: The Case of Instagram

According to IAB Spain [90], around 26 million Spaniards (representing 87% of the internet users aged between 16–65 years) are social media users. Gender profile shows a pretty balanced distribution (49% women) and the average age is around 40 years. In this sense, WhatsApp (85%) is the most used social network, followed closely by Facebook (81%), YouTube (70%) and Instagram (59%). In the ranking of users’ preferences, Instagram goes third (preferred mostly by women and the 16–24 years age segment), after WhatsApp and Facebook, while Twitter, for example, mostly preferred by men, occupies the fifth position. The abovementioned report also highlights the top three most frequently used social networks: WhatsApp (with 96% of the users operating on a daily basis), Instagram (81%) and Facebook (78%), respectively. In the same fashion, one third of the social media users indicated a higher degree of trust in brands with a social media profile, and 75% of the users mentioned that customer service is the main motive for a private interaction with a brand. Last but not least, the information collected from marketing professionals highlighted that investment in social media advertising followed an increasing trend, with Instagram as first preference, followed by Facebook and Twitter [90].

Research evidence [91,92,93,94] shows that for SMEs—bakeries, among them—social media became a relevant and attractive means for pursuing, according to Castronovo and Huang [95], at least one of the three business objectives: building awareness, increasing sales, or building loyalty. In a similar fashion, Lovejoy and Saxton [96] identified three key functions emerging from the use of Twitter by US not for profit organizations, that is: information (one-way channeling of information about the organization), community, focused on dialogue and community-building (e.g., thanks giving, recognition, supporting community events, etc.), and action (e.g., followers’ engagement with initiatives meant to help the organization such as fund raising, product selling, calls for volunteers, etc.) (pp. 343–347). According to INE [97], in Spain there are more than 3.3 million active firms. Most of them fall into the category of autonomous and professionals, with no employees (56.3%), 39.6% are small firms (one to nine employees) and 4.1% are small and medium-sized enterprises—SMEs—(10–99 employees) [97].

The Vodafone Enterprise Observatory [98], focused on the digitalization process undertaken by the professional and small firms, reported, for example, that 58% of the sample indicated that the adoption of new technologies is meant to help their business to improve customer service. In addition, 43% use social networks to advertise and push sales by attracting new customers, with Facebook (33%) as the most preferred platform, followed by Instagram (19%), Twitter (10%), and Linkedin (8%).

De Vries et al. [91], who focused on bakeries and cafes in a major urban area in New Zealand, distinguish, for example, among bakeries using the “one-way business to customer” marketing strategy to build customer basis, in social media like Facebook, while others were more engaged with their online followers through text and visual posts. Interestingly, posts opened a gate to the human dimension of the business (e.g., by disclosing “behind the scenes” actions) and had a greater impact on followers’ engagement with the bakery. Another study by Lucas and Sines [92] also found evidence of social media usage (e.g., Facebook and Instagram) for promoting sales of small businesses such as family restaurants, with specially designed marketing strategies published on Instagram. All in all, it is worthwhile to mention that an effective use of social media by SMEs is also conditioned by a proper understanding of their role as a marketing strategy tool (see [92,99]). Thus, according to the Influencer Marketing Benchmark Report 2020 [100], Instagram is the preferred social channel for the marketers interested in the B2C sector and in running influencer marketing campaigns, to increase brand awareness in the first place, followed by increasing sales. Moreover, Instagram seems to be most popular among the smaller firms, as 40% of the elicited respondents were from firms with less than ten employees and twenty 2% of them from firms with ten to fifty employees, respectively [100]. Given the growing popularity of Instagram among B2C marketers and its popularity among small firms, we focus here our attention on the use of this social channel by bakeries.

## 3. Methodology

This paper aims to understand the pandemic marketing of food through the case of bakeries. The study is based on a mixed-methods design which is focused on a series of interviews and a visual content analysis of social media. Two medium-sized cities in Catalonia, Girona, and Reus were selected to conduct the study. Both cities have around 100,000 inhabitants and they are commercial and tourist urban centers in Catalonia [101]. In order to identify and collect information from relevant sources [102,103], we used a purposeful sampling technique to select bakeries that met the following three criteria: (1) initially, a centric location, because, as according to Kowlaczyk [7] (p. 109), when it comes to geographical research, location is an important pillar towards gastronomic marketing; (2) the selected bakeries had to have more than one selling point in the city in order to assure their representation as leisure and tourism attractions within the urban environment where they are placed; (3) in addition, the bakeries had to have a social media presence, especially through Instagram, as described below. Since only three bakeries in each city met these criteria, all of them were selected and included in the research sample. Based on the data supplied by the Iberian Balance Sheet Analysis System (SABI) [104], 18% of the total Spanish businesses registered in this database either as manufacturers of bread and fresh bakery products or as retailers of bread, bakery and pastry products, are currently operating in Catalonia, almost all of them (95%), as limited liability companies (SL is the Spanish acronym) and the rest as anonymous societies (SA), except for one cooperative. According to the same source, in 2019, 95% of them did not go beyond fifty employees of which, 63% had less than ten employees and 19% between ten and twenty, respectively. The bakeries selected for the analysis fully match this profile with five of them being limited liability companies and only one, anonymous society.

First, six semi-structured interviews were conducted with a responsible manager of each of the six bakeries analyzed. The bakeries were contacted directly by the researchers and informed about the study, and therefore they were invited to participate. All of them accepted to participate and the interviews were conducted personally at their facilities during the month of November, 2020. The interviews pursued to analyze the bakeries’ performance during the lockdown and the initial phases of the recovery which started at the beginning of summer. Table 1 shows the profile of the participants which happened to be all female. As a consequence, the analysis will also provide relevant results in relation to the role of women in the management of SMEs (i.e., bakeries) during the pandemic.

In addition, in order to complement the qualitative view of the bakeries and to respond to the study aims from a quantitative perspective, their social media presence was analyzed. In particular, their Instagram accounts were selected. Instagram is an example of social media used for marketing purposes (see, for example, [105]). While other social media platforms could have been selected, the study was focused on Instagram because it is especially relevant for the promotion of gastronomy [106]. In line with previous studies, Instagram is also a prevalent source of information to conduct a content analysis based on the branding of local experiences in destinations [107]. This study applies a visual research analysis [108] based on the understanding of Albers and James, who stated that the content of a photograph is “the sum total of its appearances” [109] (p. 139). Data collection and analysis were conducted manually and included a visual codification of all the pictures posted in the six Instagram accounts (N = 638).

Instagram posts were analyzed manually to determine what type of content is offered. Each picture could include one or more objects. This allows building a more robust picture of how bakeries communicate messages online, and identifying those contents which generate a higher level of participation. The analyses included an eight-month period from March 14 (when the Spanish Government declared the state of alarm) until November 14. A pre-test analysis was performed with one of the bakeries in order to determine the category development. Four categories were defined to reflect the main elements communicated by the bakeries: product, place, people and information. Furthermore, nine subcategories were included to encapsulate the appearances in each category:

- Product: Includes the presence of bread or other products (food and beverages).
- Place: Refers to the venue, both the workshop and the selling point.
- People: The analysis differentiates bakeries’ employees and customers.
- Information: Three subcategories are developed to include congratulation days, housekeeping information and other (e.g., events or training).

Both the interviews and the analysis of social media reflect how bakeries promote a specific identity and thus a brand image, offline and online, in a pandemic environment.

## 4. Results

This section is divided into two parts. First it analyses the participants’ responses and later it discusses the features of Instagram communication.

### 4.1. Exploring Performance during Pandemic Times: A Bakeries’ Perspective

#### 4.1.1. Coping with Difficult Times: From Selling Bread to Managing People

**Figure 1:**
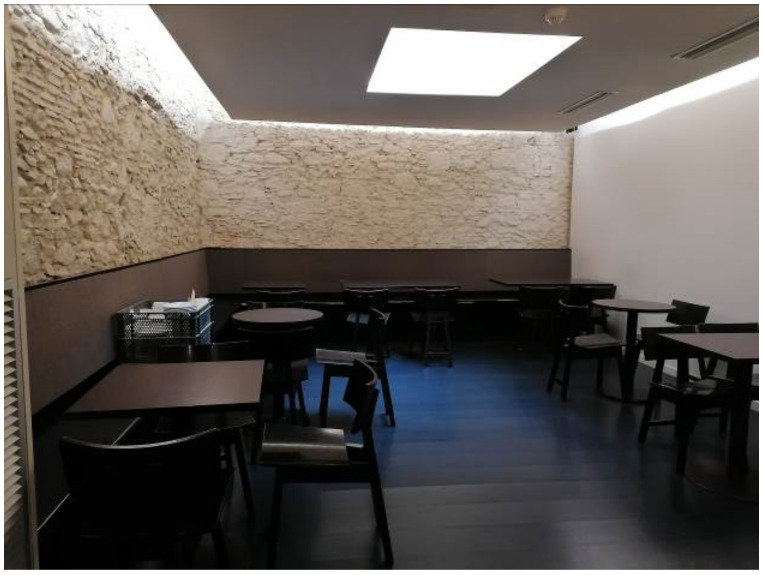
An empty cafeteria (own source).

Participants pointed out that selling bread means that they must open their facilities every day, which also includes the hardest days of the COVID-19 impact and the entire confinement period which in Spain lasted between 14 March and 21 June. However, as one of the interviewees highlighted, beyond selling bread “the cafeteria is necessary to survive”. One of the participants emphasized that “we are always open because we also sell newspapers”. But her cafeteria was obviously closed. She said that “we did not do any promotion because most customers were still coming”. While she affirms that the situation has been difficult to manage, she reported that “we increased the selling of newspapers and magazines a lot because people were at home”. However, sales did not offset the cafe. “The truth is that I would not highlight anything positive… it was a terrible three-month period and we started to breathe a little when we were able to reopen the cafeteria”. The analyzed bakeries are always open, and some of them from Monday to Sunday and from 7 a.m. to 3 p.m. A participant stated that the situation made it “difficult for people to come but it worked. We did not stop. We had to close the cafeteria but the bakery was not affected” (see, for example, Figure 1). However, since all the bakeries count on more than one store, some of them affirmed that they temporarily closed specific venues during the first months of lockdown.

Respondents obviously agreed that bread is a basic food and this forces them to be always ready to serve people. However, some of the bakeries were also urged to reduce opening schedules, and staff. “We did an ERTE (a legal mechanism that allows firms to suspend employment contracts or reduce working hours)… we kept young people but people with risk, such as older people or those living abroad, we put them in ERTE until they could rejoin…”. After the relaxation of the measures, the entire staff has been reinstated, and this was highlighted by most of the interviewees. Another participant pointed out that “we were 90 people working but after the lockdown only 75 remained because people working at the offices were the ones who went to ERTE and of course we had to close… at bakery level, sales were stable fortunately… because the cafe has had to pick up the pace again”. One interviewee mentioned that “we did not suspend contracts, some of them ended and were not renewed, but now people have rejoined if both, they and us, were happy”. In most of the cases, the whole staff has rejoined. “Everyone who wanted to come back, did it”, concluded one of the participants.

#### 4.1.2. Takeaway and Delivery to Survive

**Figure 2:**
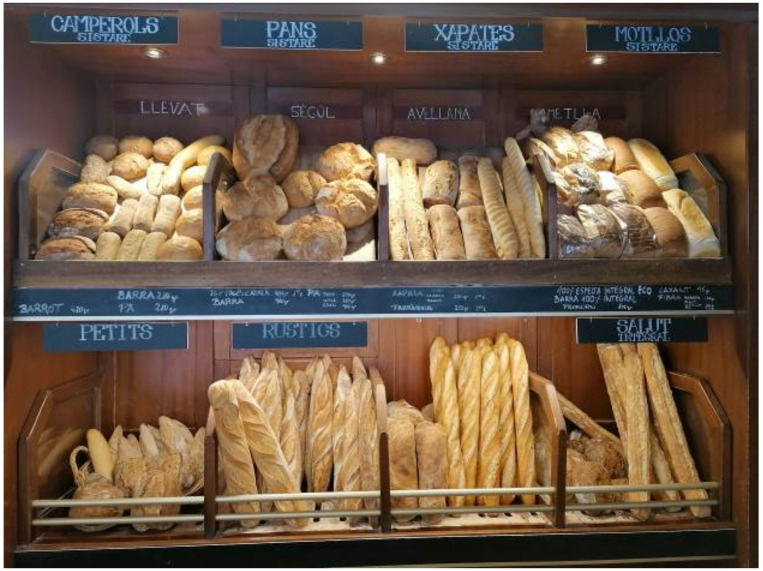
Bread products sold at bakeries (own source).

**Figure 3:**
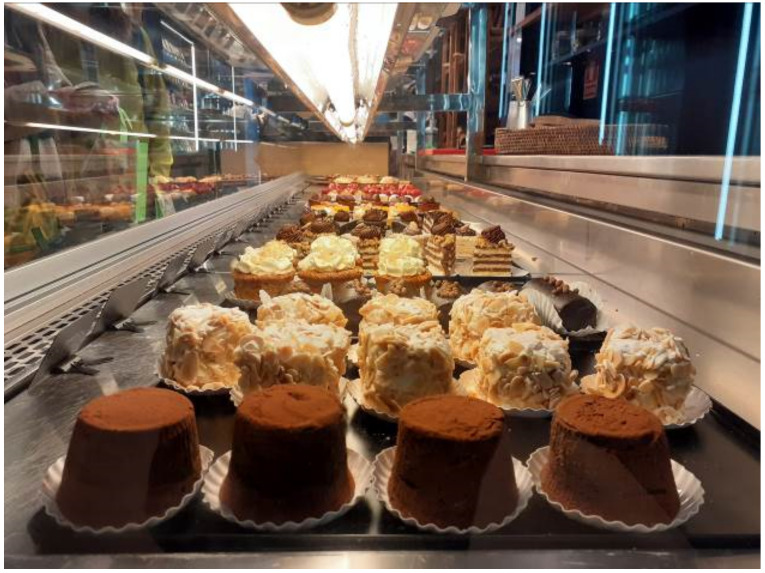
Non-bread products sold at bakeries (own source).

Although all the participants prepared takeaway food on a regular basis in their cafes, the intensification of delivery services was a key element for the survival of their firms. In some cases, they have used global platforms like Glovo or Uber. Instead, others did so by using local services. One of the interviewees underlined that she “mostly implemented takeaway and online selling in Easter, only with km0 distributors from the ‘restaurant at home’ platform”. She specifically mentioned that they would not like to use global brands, and they aim to keep their performance close to the territory and people who live in the city. Another interviewee stated that “we made home deliveries with our own delivery person who went to customers’ homes and to other stores to deliver orders”. In the second wave, started by early October, the situation was different and people were going to the selling point, which means the delivery services were reduced. The distribution included both bread products (Figure 2) and other sweets such as cakes and pastries (Figure 3).

Direct communication with customers is crucial towards the success of bakeries. One participant underlined that “especially we use the phone to take orders and delivery, not only in the city but also to small towns in the area”. Others have only maintained direct sales and have had to adapt to the new context. For example, since bars were closed this has resulted in new opportunities. One interviewee pointed out that “we were selling a lot of sandwiches. Bars were closed so we serviced a part of these customers who were looking for sandwiches” before going to work or during their breaks. It is obvious that “now when the bars reopen, sandwiches’ sales will go down and we have to make a daily forecast because it is changing a lot. Some will continue coming but others will go to the bar. But I can say there is a loyal clientele that is maintained on a daily basis”. The challenging and changing situation resulted in a lot of work that was something new for everyone and “workers had to do everything, adapt to the situation and we experienced an impressive number of orders… we had to make packages to take away at home and the employees adapted and did a very good job. We had a lot of orders and, very importantly, the customers understood the situation and respected the queues, and the rules”. This shift is further explained below.

#### 4.1.3. A Change in Production and Consumption Behaviors

The interviewees confirmed an adaptation of production derived from the situation and the changing demand. Regarding the product strategy, one participant pointed out that “we reduced the range of products and optimized what was first needed. Above all, we made large pieces, such as bread, the most essential, and we left the delicacies aside, as well as the special products”. Thus, she emphasized that they tried to meet customers’ needs and prioritized the need to the sale. While this is very important, it is not easy to understand. “The most affected people were the elderly because they did not understand that they could not buy a baguette every day. We made bigger baguettes and stopped making the smaller pieces so that they lasted for two days or more… and the older people did not understand that they could not go there to shop every day as usual…”.

**Figure 4:**
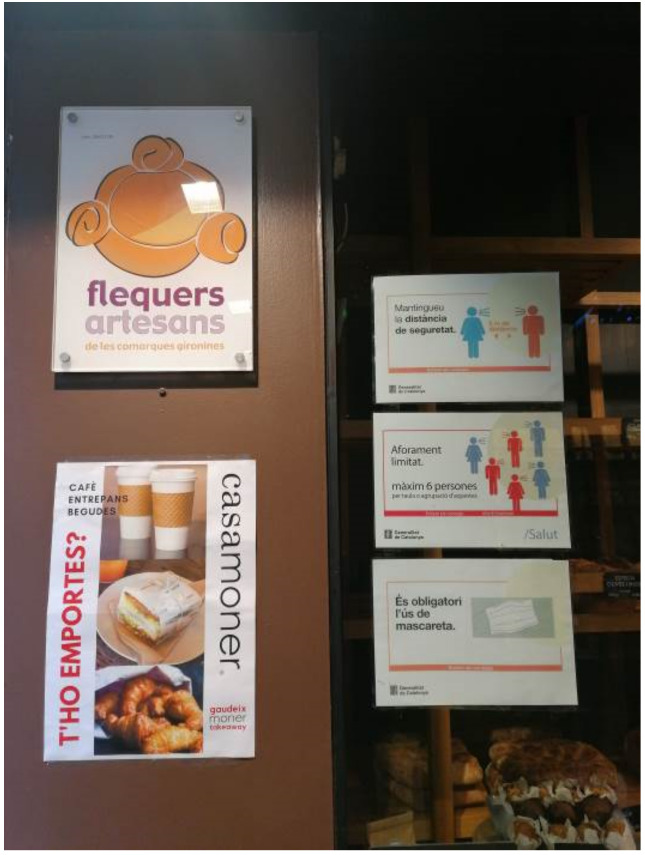
Health and service information at a bakery’s entrance (own source).

All the bakeries faced huge queues. One of the participants specifically mentioned that “when people do see queues, they think we are not affected by the virus. But we are… there are people working at the bakery, our workers, likewise customers, are affected too”. In addition, all safety measures were carried out, where a maximum of two people were allowed inside the venues, and the use of mask and hand sanitizer was compulsory (see, for example, Figure 4). Some bakeries pointed out that they already had many measures implemented before the new regulation. As mentioned above, all participants noted that consumers responded very, very well. They respected the measures and social distances and “came less but bought more. For example, they took four instead of two”. In this sense, the quality of the production is also very important for the subsequent consumption of the product. “The whole process is slow and artisanal”. One participant pointed out that “we use quality ingredients and even doctors, dietitians… have recommended our artisanal products”, which is a common feature observed in bakeries with decades of experience and even, in some cases, with more than one hundred years of history.

#### 4.1.4. Not Always Online Strategy Is the Best

In relation to the online presence of the bakeries, in some cases the interviewees stated that they have not paid much attention to online communication given the local and loyal nature of their customers. “In our case is mainly word-of-mouth but we did put few options to choose online… specially when we accepted orders for the Mona [a chocolate cake which is prepared during Easter], and customers choose whether they want it to be delivered or picked here”. Another of the participants stated that “we did not use social media”. Specially, one of the participants underlined that “we are an organic bakery with special products and a smaller assortment. We do not have a variety of offer like other bakeries but with more quality”.

In some cases; therefore, their products are sold only in the physical store because “our products are unique products that have to be explained like buckwheat bread, kamut… these are not only products, I mean you need to communicate beyond the product, and we prefer to explain directly to the buyer”. In a similar way, another participant emphasized that “we will only do it online in the long run because it is a handicap that the products cannot be made in half an hour as people ask; there are doughs that need 24, 48, 72 h, because it is a very slow product, natural, 100% organic”. This interviewee emphasized that “our strong point is the product, but our weak point is that the product is not immediate”. She continued saying “we do not have this immediacy that others can have to be able to do it online”. On the other hand, other interviewees pointed out that they did largely use “social networks to promote products and provide advice”. Additionally, a participant said “we ask for patience”, which is crucial in this complex environment. The analysis of Instagram accounts complements these reflections.

### 4.2. Profile of Social Media Promotion of Bakeries: The Role of Instagram

A visual content analysis was performed to analyze the pictures posted by the bakeries on Instagram. First of all, the number of posts they have made in the period studied is observed in Table 2. There are three bakeries that have maintained a regular activity on Instagram during the months studied, while three other bakeries have participated in social media only superficially. Additionally, most of the contributions are images.

When analyzing the number of likes and comments in Table 3, it is possible to observe the leadership of Forn Sistaré and PdePa, with an average of more than 200 likes for each post. Although in absolute terms these are low numbers, data show that bakeries are considering Instagram as a platform of communication with their current and potential customers. If the analysis focuses on the comments, here we can see a much lower participation of users, and only Antiga Casa Bellsolà and, again, Forn Sistaré stand out. In this sense, some bakeries use to publish the information along with the posts in Catalan and Spanish, and sometimes also in English, which provides evidence of the relationships between the bakeries and their customers, who are not only locals but also visitors.

In relation to the pictures with more likes and comments, we would like to highlight the three most popular posts, in particular those with more likes. There are three posts that illustrate the most liked posts from PdePa (12 May 2020, 820 likes), Antiga Casa Bellsolà (7 April 2020, 727 likes) and Forn Sistaré (26 October 2020, 518 likes). The ranking of posts based only on comments is not presented because the post with the greatest number of comments includes only 15, which represents a very low online interaction between the bakeries and their customers.

The first post (PdePA Coffee Bakery (@pdepabakery), posted in 12 May 2020) is published with a text where the bakery says “after a morning of work, it’s time to take a break. What if you give yourself a PdePa pizza?” In addition, the post shows all the alternatives for orders and delivery so that customers can buy the product and eat it either at work or at home. At the beginning of May, in Spain there was still a state of alarm, but the strictest confinement was beginning to relax. While this bakery has continuously reminded takeaway and delivery options (by phone call or e-commerce), it was also important to provide a safe environment when cafeterias were reopened. In this sense, they published a post to inform that: “At PdePA we care about both your safety and that of our team. That’s why we test our workers on Covid19 before coming to work” (@pdepabakery, 15 May 2020).

The second post (Antiga Casa Bellsolà (@antigacasabellsola), posted in 7 April 2020), on the other hand, was published during the most restrictive weeks, and the bakery reported on home delivery and highlighted its contribution to local food systems. The post says “we bring you artisan bread and our freshly made products to your home. These days, more than ever, eat well, enjoy quality and support local trade”. In this sense, there are a lot of posts that announce the availability of takeaway and delivery services, or the implementation of new measures such as the use of QR codes. In particular, this publication encourages customers to make collaborative orders: “And we remind you that you can arrange a joint order with neighbors and reach the minimum purchase of € 20 for home delivery. So, all of you will enjoy, without leaving home, quality products, artisanal, freshly made and of proximity trade. Call for orders. We serve about 10km around Girona” (@antigacasabellsola, 23 April 2020).

The uncertainty of the second wave had implied a series of restrictive measures in some regions (Catalonia had closed cafeterias and restaurants during one month between mid-October and mid-November). Some posts published by different bakeries inform again about the new situation: “Dear customers, due to the new restrictions to deal with Covid19, from tomorrow Friday we can only serve you to take away. You will find the same assortment every day for breakfast, lunch and snack but you will not be able to enjoy it in our facilities. We hope that in 15 days we can return to the normality we had but, above all, we hope that you all take great care and stay very healthy. These are not easy times and our entire industry is making big efforts in order to be able to stay afloat while trying to control the virus. We do not know what more will be required to us and we do not know how long this coming and going of measures will last, so we ask you to support the sector while you intensify precautionary measures to contain Covid19. What we do know is that if we all aim to, we will get out of it soon. Good health to everyone and thank you for being there” (@roslenamoments, 15 October 2020).

Finally, the third post (Forn Sistaré (@fornsistare), posted on 26 October 2020) refers to a post published in the month of October. In particular, the post showcases the participation of Forn Sistaré, in a set of four photographs, in a Spanish television program about artisan bread. In this sense, this bakery has also thanked on different occasions their customers for their understanding. This post illustrates it: “Thanks! Thank you for your public spirit, patience and trust. This time, in the face of what we have to live, each one has a role to play. We, as bakers, continue to work to supply one of the most basic foods that has ever existed, but now, more than ever, it is clear that origin attributable to PA [bread in Catalan language], that of comPArtir [share]. To minimize risks and avoid complications we have had to close some stores, and we understand that for some of you it can be an added inconvenience. Now you must stay home even if it’s not strictly necessary. Only by sharing these values and the confinement we will succeed” (@fornsistare, 28 March 2020).

This said, one of the most important elements that contribute to the construction of the identity that bakeries create in the digital environment is the content included in the posts. In Table 4 we can observe that most of the content refers to the product, with more than half of the representations. The other half is divided between the shop and production spaces, people (both bakeries’ employees and customers) and the information they communicate through Instagram. A total of 638 images with 738 representations were analyzed.

After the data analysis, Table 5 reveals the results according to the different sub-categories, and with regards to each bakery. Although bread has a prominent presence (for example, the description and promotion of special breads as published by @antigacasabellsola in July 22 or September 17), the category of other products far exceeds the presence of own bread. In this sense, other products include cakes and pastries (as displayed above), but also pizzas and other savory products, as well as drinks, such as coffee or slushes in summer. Visual representations of foods appear in both the shop and the counters, but also as takeaway options.

On the other hand, the place is mainly promoted through the shop itself, showing the decorated doors and windows, the machinery like the coffee makers, the inner tables, and the outer terraces. All the space where the shopping experience takes place is widely represented. At the same time, in some cases, such as Antiga Casa Bellsolà and Forn Sistaré, the workshop is also shown, where a significant marketing role is given to the artisanal processes, such as the preparation of the dough.

In relation to people, in this category the posts include mainly bakery workers, which include bakers and front-line staff as well as employees and waiters. With a more limited presence, some pictures also represent distributors. Customers are also included under the category other people, as they buy and consume. Additionally, especially during confinement, people are photographed eating the products outdoors. Once the terraces were reopened, the clientele was spotted eating in the bakeries’ terraces.

Finally, in relation to the theme of information, this category serves as an avenue to communicate further with customers. In this sense, bakeries take the opportunity to provide information on opening times or products. Also, there are some posts to congratulate special days such as Sant Joan or local festivities. As explained in the methodology section, the other subcategory includes various elements with a low representation. With regards to the special days, Forn Sistaré published a post on the World Bread Day (October 16) where they acknowledge the value of making bread and also wish health, prudence and optimism to people: “For us it is a day to highlight and make known a little more this profession that we love so much. Making artisan bread for us is a way of life, a way of understanding the territory, food and relationships... and pedagogy and communication. We have a commitment to our customers, our neighbors and society, and it is our job to explain and teach how to make artisan bread, how we relate to farmers, suppliers, other bakers, and how it affects food and health..., also a great world to explore the whole issue of intolerance. We celebrate every day that we can enjoy good artisan and healthy bread! Health, prudence and optimism!” (@fornsistare, 16 October 2020).

## 5. Discussion and Conclusions

The role of food in leisure and tourism has experienced a growing interest in recent decades. A wide range of culinary attractions and practices have been progressively placed at the center of leisure and tourist experiences and destination promotion. According to Freire and Gertner [54], food is a key destination brand dimension. In particular, this paper analyzed the performance of a specific branch of city food services and a specific attraction of food tourism systems: the bakeries. After a first analysis carried out via interviews made with representatives of the six bakeries, results confirm a series of implications. All the participants have explained to the authors that the situation derived from the spread of the COVID-19 has challenged them both as individuals and workers. The measures implemented by the Spanish government have also challenged the bakeries they work at. In general, the intensification of takeaway and delivery options and the temporarily reduction of staff have been the main strategies that bakeries have followed to cope with the pandemic context. From a marketing perspective, the online presence, although it is still not widespread, has been in some cases key to maintaining a constant and immediate dialogue with customers. This has been observed in three of the six bakeries. Therefore, the digital environment still offers many opportunities because all the participants highlighted that daily relationships between bakeries and customers are developed via a direct communication, which is facilitated because they sell a staple product such as bread. Thus, given the heavy territorial roots of the bakeries, the interviewees emphasized the importance of personal communication with both employees and customers in order to maintain the identity of the bakeries in uncertain and complex times.

Bakeries were forced to reduce or suspend the contracts of some of their employees, especially those working in the cafeteria service. Cafeteria tends to be the main source of income for bakeries, and this was completely stopped during the lockdown period. However, bakeries kept a fluent communication with their customers directly at selling points. The bakeries have also faced a change in consumption patterns which led to a shift in production, for example, a focus on larger pieces of bread or an intensive elaboration of sandwiches to replace the lack of service provided by closed bars. In a rapid process of change, the bakeries started or expanded their takeaway and delivery services. Different strategies were observed, but most of the participants showed resilience and adjusted their offers to meet the demand and supply their products to their clientele. To achieve this, a direct communication is mandatory. While all the participants acknowledged the added value of personal communication with customers (at the own shop or by phone), social media was also mentioned as a pathway to keep current and potential clients up to date. However, this was not a general trend. While previous research reveals the role of social media as strategy to interact between firms and customers, and build loyalty (see, for example, [91]), results of this study confirm that bakeries (as SMEs in a Catalan context) primarily use social media to increase sales due to economic and time constraints and they generate very little conversation. This means that there is still much room to improve the implementation of social media to co-create experiences and engage with customers in a more active way to enhance mutual communication and feedback.

In this sense, for those using Instagram as a regular marketing strategy, the visual analysis showcases that posts included a virtual narrative focused on the product. This does not only promote bread, but also other products they elaborate. Specially, the variety of products account for more than half of total objects studied. In addition, both the place and the people represent significant objects that construct the digital identity of the bakeries. Their social media profiles show that they are attached to the city and the territory where they are located. The territory influences them (for example, using basic ingredients to make bread) but they also influence the territory and its people. As explained above, the way the bakeries promote themselves (offline and online) shows how they are. What is the experience the bakeries create? A passion for service in pandemic times, and a passion for the elaboration of products, and the subsequent tasting by customers—giving sense to the production processes. This is the identity experience offered by the bakeries, and one of the main theoretical implications of this research. Branding is an instrument to obtain competitive advantage [62] and the brand image contributes to provide unique experiences, and consequently generate positive word-of-mouth (see, for example, [110]) and electronic word-of-mouth (see, for example, [111]).

Since this study relies on a limited sample focused only on two cities in the Catalan context, north-eastern Spain, results of this research cannot be generalized to the overall situation of the sector. Thus, the paper can be a starting point for further research that also examines the nature of the users’ comments and consider the social media marketing strategies in other platforms such as Facebook, Twitter, and Tik Tok which would reveal more robust insights into the use of social media in destination branding [112] and allow comparison between different platforms [113]. Previous research shows that social media is an effective strategy to communicate brand identity and influence customers [80], and even change customers’ behaviors [114]. However, it is important to consider that marketing local food products also refer to different types of visitors [65], an aspect which requires further analysis. In this sense, some authors stated that food and beverage tasting is an aesthetic experience [115,116], where induced images play a vital role in co-marketing [117]. This is a main practical implication of this research. The integration of food heavily contributes to the development of a place image [118], and this research confirms that bakeries play a meaningful part in destination branding because of the relevance of food for destination management and marketing.

Further research also needs to delve into the placement of bakeries within the entire gastronomy landscapes of a destination, and specially within the promotion of food by local and regional DMO’s and how stakeholders’ efforts may lead to the attraction of food visitors [78]. Branding and marketing strategies must focus on the potential of food tourism drawing on: Proudness of local food, empowerment of local SMEs, and development of synergies among local food products and services [38], which also inform a sustainable food consumption strategy (see [119,120]).

Furthermore, according to Buhalis and Sinarta [77], “social media can be used as a real-time interactive channel to co-create value with consumers” (p. 564), a trend that bakeries, as examples of SMEs, should also follow. This would not only improve customer support but also personalized experiences. Instant services are critical in the new service experiences, and to survive in a changing environment. Real-time marketing adds value to brands and here bakeries need to place “mechanisms to establish dynamic engagement with consumers and offer agile and context-based services” [77] (p. 579). Further research will provide a more robust understanding of branding of food leisure and tourism stakeholders by expanding the type of venues and the geographical regions studied. In addition, since the study sample is formed only by women, future studies could also approach the analysis of food and tourism experiences from a gender perspective. Further research opportunities also emerge from the analysis of the potential negative impact of the social media (e.g., ratings) on brands’ image, as well as, of the relationships between food tourism, crisis management and service experiences where online and offline branding and marketing are crucial to the communication of food identity, business identity, and destination identity.

## Figures and Tables

**Table 1:** Profile of the interviewees.

Participant	Genre	Age	Experience Working at the Bakery	Education
1	Female	60	7	Vocational studies
2	Female	53	10	Bachelor
3	Female	60	30	Secondary school
4	Female	42	1	Master
5	Female	25	7	Vocational studies
6	Female	33	7	Bachelor

**Table 2:** Number of posts and images (and videos) of bakeries on Instagram.

Bakery	Posts	Pictures	Videos
Antiga Casa Bellsolà	131	127	8
Casa Moner	4	5	0
Fleca Flaqué	30	35	0
Forn Sistaré	121	151	19
PdePa	268	282	1
Roslena	35	38	1
Total	589	638	29

**Table 3:** Likes and comments of bakeries’ posts on Instagram.

Bakery	Likes	Likes/Picture	Comments	Comments/Picture
Antiga Casa Bellsolà	16,301	132,53	546	4,44
Casa Moner	425	106,25	7	1,75
Fleca Flaqué	218	7,27	6	0,2
Forn Sistaré	23,449	229,89	358	3,51
PdePa	58,887	220,55	313	1,17
Roslena	2095	61,62	34	1

**Table 4:** Totals of categories represented on bakeries’ Instagram.

	Product	Place	People	Information	Total
N	413	118	113	94	738
%	56%	16%	15%	13%	100%

**Table 5:** The categories and sub-categories represented on the bakeries’ Instagram.

	Product		Place		People		Information			Total
	Bread	Other Food	Shop	Workshop	Employees	Other People	Congratulations	Housekeeping Information	Other	
Antiga Casa Bellsolà	37	42	24	10	5	5	3	17	2	145
Casa Moner		3		1						4
Fleca Flaqué	6	17	10		2					35
Forn Sistaré	19	47	5	9	13	8	10	14	12	137
PdePa	16	203	56		41	37	4	15	4	376
Roslena	5	18	3		1	1	3	1	9	41
Total	83	330	98	20	62	51	20	47	27	738

## Data Availability

Data is contained within the article.

## References

[1] Henderson J.C. (2009). Food tourism reviewed. Br. Food J..

[2] Hall C.M., Timothy D. (2016). Heirloom products in heritage places: Farmers markets, local food, and food diversity. Heritage Cuisines: Traditions, Identities and Tourism.

[3] Europa Press (2019). El 40% del Gasto Turístico se Destina a Gastronomía. Europa Press. https://www.europapress.es/turismo/nacional/noticia-40-gasto-turistico-destina-gastronomia-20190213085935.html.

[4] KPMG (2019). La Gastronomía en la Economía Española.

[5] Wolf E. (2014). Have Fork Will Travel: A Practical Handbook for Food and Drink Tourism Professionals.

[6] Panatics Association (2021). La Ruta del Buen Pan. https://panatics.com/eventos/la-ruta-del-buen-pan.

[7] Kowlaczyk A., Kowlaczyk A., Derek M. (2020). Dimensions of Gastronomy in Contemporary Cities. Gastronomy and Urban Space. Changes and Challenges in Geographical Perspective.

[8] Hall C.M., Gössling S. (2016). Food Tourism and Regional Development: Networks, Products and Trajectories.

[9] Gössling S., Scott D., Hall C.M. (2021). Pandemics, tourism and global change: A rapid assessment of COVID-19. J. Sustain. Tour..

[10] Newsome D. (2020). The collapse of tourism and its impact on wildlife tourism destinations. J. Tour. Futures.

[11] Williams C.C. (2020). Impacts of the coronavirus pandemic on Europe’s tourism industry: Addressing tourism enterprises and workers in the undeclared economy. Int. J. Tour. Res..

[12] UNWTO (2020). International Tourism and COVID-19. https://www.unwto.org/international-tourism-and-covid-19.

[13] Ministry of Industry, Trade and Tourism (2020). COVID-19 News. https://www.mincotur.gob.es/en-us/COVID-19/Paginas/COVID-19.aspx.

[14] National Institute of Statistics, INE (2021). Tourist Movement on Borders Survey Frontur. https://www.ine.es/dyngs/INEbase/es/operacion.htm?c=Estadistica_C&cid=1254736176996&menu=ultiDatos&idp=1254735576863.

[15] National Institute of Statistics, INE (2021). Tourist Expenditure Survey Egatur. https://www.ine.es/dyngs/INEbase/es/operacion.htm?c=Estadistica_C&cid=1254736177002&menu=ultiDatos&idp=1254735576863.

[16] Rodríguez-Antón J.M., Alonso-Almeida M.M. (2020). COVID-19 Impacts and Recovery Strategies: The Case of the Hospitality Industry in Spain. Sustainability.

[17] Hortoinfo (2021). The Agricultural Sector Has Been the Only One in Spain That Increased GDP in a 2020 Pandemic (26 March 2021). http://www.hortoinfo.es/index.php/10291-pib-sector-agricola-260321.

[18] Montoriol-Garriga J. (2020). The Strength of the Agrifood Sector during the Coronavirus Crisis. CaixaBank Research. https://www.caixabankresearch.com/en/sector-analysis/agrifood/strength-agrifood-sector-during-coronavirus-crisis?index.

[19] Montoriol-Garriga J. (2020). Changing Consumption Patterns during Lockdown: From the Restaurant to the Home. CaixaBank Research. https://www.caixabankresearch.com/en/sector-analysis/agrifood/changing-consumption-patterns-during-lockdown-restaurant-home.

[20] CIS (2020). Centre of Sociological Research. Barometer, May 2020. http://www.cis.es/cis/opencm/EN/11_barometros/index.jsp.

[21] Laguna L., Fiszman S., Puerta P., Chaya C., Tárrega A. (2020). The impact of COVID-19 lockdown on food priorities. Results from a preliminary study using social media and online survey with Spanish consumers. Food Qual. Prefer..

[22] Everett S., Aitchison C. (2008). The role of food tourism in sustaining regional identity: A case study of Cornwall, South West England. J. Sustain. Tour..

[23] Sims R. (2009). Food, place and authenticity: Local food and the sustainable tourism experience. J. Sustain. Tour..

[24] Bessière J. (1998). Local development and heritage: Traditional food and cuisine as tourist attractions in rural areas. Eur. Soc. Rural Sociol..

[25] Cusack I. (2000). African cuisines: Recipes for nation building?. J. Afr. Cult. Stud..

[26] Du Rand G.E., Heath E. (2006). Towards a framework for food tourism as an element of destination marketing. Curr. Issues Tour..

[27] Rousta A., Jamshidi D. (2019). Food tourism value: Investigating the factors that influence tourists to revisit. J. Vacat. Mark..

[28] Fusté-Forné F., Berno T. (2016). Food tourism in New Zealand: Canterbury’s foodscapes. J. Gastron. Tour..

[29] Nelson V. (2016). Food and image on the official visitor site of Houston, Texas. J. Destin. Manag. Mark..

[30] Carpenter S., Takahashi B., Cunningham C., Lertpratchya A. (2016). The roles of Social media in promoting sustainability in higher education. Int. J. Commun..

[31] Okumus B. (2020). Food tourism research: A perspective article. Tour. Rev..

[32] Amick B. (2018). Food tourism benefits bakeries. https://www.bakemag.com/articles/6088-food-tourism-benefits-bakeries.

[33] Long L.M. (2003). Culinary Tourism.

[34] Hall C.M., Sharples L., Hall C.M., Sharples L., Mitchell R., Macionis N., Cambourne B. (2003). The consumption of experiences or the experiences of consumption? An introduction to the tourism of taste. Food Tourism: Around the World: Development, Management and Markets.

[35] Ellis A., Park E., Kim S., Yeoman I. (2018). What is food tourism?. Tour. Manag..

[36] Fusté-Forné F., Mundet L. (2020). A land of cheese: From food innovation to tourism development in rural Catalonia. J. Tour. Cult. Chang..

[37] Stone M.J., Migacz S., Wolf E. (2018). Beyond the journey: The lasting impact of culinary tourism activities. Curr. Issues Tour..

[38] Du Rand G.E., Heath E., Alberts N. (2003). The role of local and regional food in destination marketing: A South African situation analysis. J. Travel Tour. Mark..

[39] Knollenberg W., Duffy L.N., Kline C., Kim G. (2020). Creating Competitive Advantage for Food Tourism Destinations Through Food and Beverage Experiences. Tour. Plan. Dev..

[40] Fusté-Forné F. (2020). Savouring place: Cheese as a food tourism destination landmark. J. Place Manag. Dev..

[41] Amore A., Roy H. (2020). Blending foodscapes and urban touristscapes: International tourism and city marketing in Indian cities. Int. J. Tour. Cities.

[42] Andersson T.D., Mossberg L., Therkelsen A. (2017). Food and tourism synergies: Perspectives on consumption, production and destination development. Scand. J. Hosp. Tour..

[43] Choe J.Y., Kim J.H., Cho M.S. (2017). A comparison of food contents used by official tourism organizations’ mobile applications. J. Gastron. Tour..

[44] Okumus F., Kock G., Scantlebury M.M., Okumus B. (2013). Using local cuisines when promoting small Caribbean island destinations. J. Travel Tour. Mark..

[45] Chan A.P.H., Tung V.W.S. (2019). Examining the effects of robotic service on brand experience: The moderating role of hotel segment. J. Travel Tour. Mark..

[46] Murphy J., Gretzel U., Pesonen J. (2019). Marketing robot services in hospitality and tourism: The role of anthropomorphism. J. Travel Tour. Mark..

[47] McKercher B. (2020). Cultural tourism market: A perspective paper. Tour. Rev..

[48] Kim Y.H., Yuan J., Goh B.K., Antun J.M. (2009). Web marketing in food tourism: A content analysis of web sites in West Texas. J. Culin. Sci. Technol..

[49] Marzano G., Scott N. (2009). Power in destination branding. Ann. Tour. Res..

[50] Perkins R., Khoo-Lattimore C., Arcodia C. (2020). Understanding the contribution of stakeholder collaboration towards regional destination branding: A systematic narrative literature review. J. Hosp. Tour. Manag..

[51] Morgan N., Pritchard A., Pride R. (2007). Destination Branding.

[52] Rather R.A., Najar A.H., Jaziri D. (2020). Destination branding in tourism: Insights from social identification, attachment and experience theories. Anatolia.

[53] Pike S. (2005). Tourism destination branding complexity. J. Prod. Brand Manag..

[54] Freire J.R., Gertner R.K. (2021). The relevance of food for the development of a destination brand. Place Branding Public Dipl..

[55] Lai M.Y., Khoo-Lattimore C., Wang Y. (2019). Food and cuisine image in destination branding: Toward a conceptual model. Tour. Hosp. Res..

[56] Campelo A., Aitken R., Thyne M., Gnoth J. (2014). Sense of place: The importance for destination branding. J. Travel Res..

[57] Lai M.Y., Khoo-Lattimore C., Wang Y. (2018). A perception gap investigation into food and cuisine image attributes for destination branding from the host perspective: The case of Australia. Tour. Manag..

[58] Lin Y.C., Pearson T.E., Cai L.A. (2011). Food as a form of destination identity: A tourism destination brand perspective. Tour. Hosp. Res..

[59] Lee I., Arcodia C. (2011). The role of regional food festivals for destination branding. Int. J. Tour. Res..

[60] Yang F.X., Wong I.A., Tan X.S., Wu D.C.W. (2020). The role of food festivals in branding culinary destinations. Tour. Manag. Perspect..

[61] Kavaratzis M. (2009). Cities and their brands: Lessons from corporate branding. Place Branding Public Dipl..

[62] Zhang L., Zhao S. (2009). City branding and the Olympic effect: A case study of Beijing. Cities.

[63] Sahin S., Baloglu S. (2014). City Branding: Investigating a brand advocacy model for distinct segments. J. Hosp. Mark. Manag..

[64] Baker B. (2007). Destination Branding for Small Cities: The Essentials for Successful Place Branding.

[65] McKercher B., Okumus F., Okumus B. (2008). Food tourism as viable market segment: It’s all how you cook the numbers. J. Travel Tour. Mark..

[66] Sørensen F., Jensen J.F. (2015). Value creation and knowledge development in tourism experience encounters. Tour. Manag..

[67] Tsai C., Wang Y. (2017). Experiential value in branding food tourism. J. Destin. Mark. Manag..

[68] Brakus J.J., Schmitt B.H., Zarantonello L. (2009). Brand experience: What is it? How is it measured? Does it affect loyalty?. J. Mark. Res..

[69] Marlowe B., Lee S. (2018). Conceptualizing terroir wine tourism. Tour. Rev. Int..

[70] Vrasida M., Peistikou M., Iliopoulou N., Kavoura A., Kefallonitis E., Theodoridis P. (2020). Developing a Tourism Destination Through Gastronomy Branding. Strategic Innovative Marketing and Tourism.

[71] Qu H., Kim L.H., Im H.H. (2011). A model of destination branding: Integrating the concepts of the branding and destination image. Tour. Manag..

[72] Ryu K., Lee H.R., Kim W.G. (2012). The influence of the quality of the physical environment, food and service on restaurant image, customer perceived value, customer satisfaction, and behavior intentions. Int. J. Contemp. Hosp. Manag..

[73] Wu H.-C. (2013). An empirical study of the effects of service quality, perceived value, corporate image, and customer satisfaction on behavioral intentions in the Taiwan quick service restaurant industry. J. Qual. Assur. Hosp. Tour..

[74] Choe J.Y.J., Kim S.S. (2018). Effects of tourists’ local food consumption value on attitude, food destination image, and behavioral intention. Int. J. Hosp. Manag..

[75] Mkono M., Tribe J. (2017). Beyond reviewing: Uncovering the multiple roles of tourism social media users. J. Travel Res..

[76] Buhalis D., Foerste M. (2015). SoCoMo marketing for travel and tourism: Empowering co-creation of value. J. Destin. Mark. Manag..

[77] Buhalis D., Sinarta Y. (2019). Real-time co-creation and nowness service: Lessons from tourism and hospitality. J. Travel Tour. Mark..

[78] Mohammad Arif A.S., Du J.T. (2019). Understanding collaborative tourism information searching to support online travel planning. Online Inf. Rev..

[79] Xiang Z., Gretzel U. (2010). Role of social media in online travel information search. Tour. Manag..

[80] Bu Y., Parkinson J., Thaichon P. (2020). Digital content marketing as a catalyst for e-WOM in food tourism. Australas. Mark. J..

[81] Naidoo V., Hollebeek L.D. (2016). Higher education brand alliances: Investigating consumers’ dual-degree purchase intentions. J. Bus. Res..

[82] Jefferson S., Tanton S. (2015). Valuable Content Marketing: How to Make Quality Content Your Key to Succes.

[83] Ashley C., Tuten T. (2015). Creative strategies in social media marketing: An exploratory study of branded social content and consumer engagement. Psychol. Mark..

[84] Westbrook R.A. (1987). Product/consumption based affective responses and post-purchase processes. J. Mark. Res..

[85] Litvin S.W., Goldsmith R.E., Pan B. (2008). Electronic word-of-mouth in hospitality and tourism management. Tour. Manag..

[86] Effendi M.I., Sugandini D., Istanto Y. (2020). Social Media Adoption in SMEs Impacted by COVID-19: The TOE Model. J. Asian Financ. Econ. Bus..

[87] Saroj A., Pal S. (2020). Use of social media in crisis management: A survey. Int. J. Disaster Risk Reduct..

[88] Pasquinelli C., Trunfio M., Bellini N., Rossi S. (2021). Sustainability in overtouristified cities? A social media insight into Italian branding responses to COVID-19 crisis. Sustainability.

[89] Priporas C.V., Stylos N., Kamenidou I.E. (2020). City image, city brand personality and generation Z residents’ life satisfaction under economic crisis: Predictors of city-related social media engagement. J. Bus. Res..

[90] Iab Spain (2020). Social Networks Study 2020. https://iabspain.es/presentacion-estudio-redes-sociales-2020.

[91] De Vries H.P., Veer E., De Vries K.V. (2018). An examination of SME social media use in the food industry. Small Enterp. Res..

[92] Lucas T., Sines C.C. (2019). Marketing strategies to increase sales in small family-style retaurant businesses. J. Soc. Media Soc..

[93] Meske C., Stieglitz S., Harmsen F., Proper H.A. (2013). Adoption and Use of Social Media in Small and Medium-Sized Enterprises. Practice-Driven Research on Enterprise Transformation.

[94] Schaupp L.C., Bélanger F. (2014). The value of social media for small businesses. J. Inf. Syst..

[95] Castronovo C., Huang L. (2012). Social media in an alternative marketing communication model. J. Mark. Dev..

[96] Lovejoy K., Saxton G.D. (2012). Information, community, and action: How nonprofit organizations use social media. J. Comput. Mediat. Commun..

[97] INE (2021). National Institute of Statistics. https://www.ine.es.

[98] VEO (2019). The Vodafone Enterprise Observatory 2019. Professionals and Small Firms. https://xh4y28w4m30fiwf22ex7gvfa-wpengine.netdna-ssl.com/wp-content/uploads/2019/11/OVE_-III-Estudio-sobre-el-Estado-de-la-Digitalizacio%CC%81n-Microempresas-2019.pdf.

[99] Li X. (2012). Weaving social media into a business proposal project. Bus. Commun. Q..

[100] Influencer Marketing Hub (2021). Influencer Marketing Benchmark Report 2020. https://influencermarketinghub.com/influencer-marketing-benchmark-report-2020.

[101] IDESCAT (2020). Població. https://www.idescat.cat/tema/xifpo.

[102] Cresswell J.W., Plano Clark V.L. (2011). Designing and Conducting Mixed Method Research.

[103] Patton M.Q. (2002). Qualitative Research and Evaluation Methods.

[104] SABI (2021). The Iberian Balance Sheet Analysis System. Bureau van Dijk. Universitat de Girona, Spain. https://biblioteca.udg.edu/ca.

[105] Fatanti M.N., Suyadnya I.W. (2015). Beyond user gaze: How Instagram creates tourism destination brand?. Procedia Soc. Behav. Sci..

[106] Vila M., Costa G., Ellinger E. (2021). An ethnographic study of the motivations of foodstagrammer tourists. J. Sustain. Tour..

[107] Gon M. (2021). Local experiences on Instagram: Social media data as source of evidence for experience design. J. Destin. Mark. Manag..

[108] Spencer S. (2010). Visual Research Methods in the Social Sciences: Awakening Visions.

[109] Albers P.C., James W.R. (1988). Travel photography: A methodological approach. Ann. Tour. Res..

[110] Papadimitriou D., Kaplanidou K., Apostolopoulou A. (2018). Destination image components and word-of-mouth intentions in urban tourism: A multigroup approach. J. Hosp. Tour. Res..

[111] Chen Y.F., Law R. (2016). A review of research on electronic word-of-mouth in hospitality and tourism management. Int. J. Hosp. Tour. Adm..

[112] Ruiz-Real J.L., Uribe-Toril J., Gázquez-Abad J.C. (2020). Destination branding: Opportunities and new challenges. J. Destin. Mark. Manag..

[113] Lu W., Stepchenkova S. (2015). User-generated content as a research mode in tourism and hospitality applications: Topics, methods, and software. J. Hosp. Mark. Manag..

[114] Leung X.Y., Sun J., Bai B. (2019). Thematic framework of social media research: State of the art. Tour. Rev..

[115] Charters S., Pettigrew S. (2005). Is wine consumption an aesthetic experience?. J. Wine Res..

[116] Koponen S., Mustonen P. (2020). Eating alone, or commensality redefined? Solo dining and the aestheticization of eating (out). J. Consum. Cult..

[117] Vanolo A. (2007). City Branding: The Ghostly Politics of Representation in Globalising Cities.

[118] Tellström R., Gustafsson I.B., Mossberg L. (2006). Consuming heritage: The use of local food culture in branding. Place Branding.

[119] Martínez-Navalón J.G., Gelashvili V., Saura J.R. (2020). The Impact of Environmental Social Media Publications on User Satisfaction with and Trust in Tourism Businesses. Int. J. Environ. Res. Public Health.

[120] Fennell D.A., Bowyer E. (2020). Tourism and sustainable transformation: A discussion and application to tourism food consumption. Tour. Recreat. Res..

